# The Antiobesity Effect of *Polygonum aviculare* L. Ethanol Extract in High-Fat Diet-Induced Obese Mice

**DOI:** 10.1155/2013/626397

**Published:** 2013-01-29

**Authors:** Yoon-Young Sung, Taesook Yoon, Won-Kyung Yang, Seung Ju Kim, Dong-Seon Kim, Ho Kyoung Kim

**Affiliations:** ^1^Basic Herbal Medicine Research Group, Korea Institute of Oriental Medicine, Daejeon 305-811, Republic of Korea; ^2^Herbal Material Management Team, Korea Institute of Oriental Medicine, Daejeon 305-811, Republic of Korea

## Abstract

The antiobesity effects of a *P. aviculare* ethanol extract (PAE) in high-fat diet- (HFD-) induced obese mice were investigated. The mice were fed an HFD or an HFD supplemented with PAE (400 mg/kg/day) for 6.5 weeks. The increased body weights, adipose tissue weight, and adipocyte area as well as serum total triglyceride, leptin, and malondialdehyde concentrations were decreased in PAE-treated HFD-induced obese mice relative to the same measurements in untreated obese mice. Furthermore, PAE significantly suppressed the elevated mRNA expression levels of sterol regulatory element-binding protein-1c, peroxisome proliferator-activated receptor **γ**, fatty acid synthase, and adipocyte protein 2 in the white adipose tissue of obese mice. In addition, PAE treatment of 3T3-L1 cells inhibited adipocyte differentiation and fat accumulation in a dose-dependent manner. These results suggest that PAE exerts antiobesity effects in HFD-induced obese mice through the suppression of lipogenesis in adipose tissue and increased antioxidant activity.

## 1. Introduction

Obesity accelerates the accumulation of excess fat, which is associated with numerous metabolic disorders, including hyperlipidemia, diabetes mellitus, hypertension, atherosclerosis, cardiovascular disease, and certain types of cancer; all of these conditions can lead to further morbidity and mortality [1]. To treat and control obesity, pharmacological approaches such as drugs to induce loss of appetite, inhibit nutrient absorption, and promote weight loss have been used [2]. Clinically available antiobesity agents include orlistat, a pancreatic lipase inhibitor, and sibutramine, a centrally acting inhibitor of serotonin and norepinephrine uptake [3, 4]. However, these antiobesity agents cause serious adverse effects, including constipation, insomnia, emesis, headache, stomachache, and myocardial infarction [5]. Bangpoongtongsungsan (BPT), a traditional herbal medicine composed of 18 crude drugs, has been used as an antiobesity drug in overweight patients [6]. In high-fat diet-(HFD-) fed mice, BPT decreased the weight of white adipose tissue and size of adipocytes [7]. At present, many oriental medicinal herbs are reported to effectively treat obesity without significant side effects and might be an excellent alternative strategy to develop safe and effective antiobesity drugs [8, 9].

*Polygonum aviculare* L. (knotgrass), a member of the Polygonaceae family, has traditionally been used as a diuretic, astringent, insecticide, and antihypertensive [10, 11]. The plant is used to treat arthritis, gout, hemorrhage, diarrhea, dysentery, hemoptysis, and hemorrhoids [11]. Reports suggest that *P. aviculare* extracts induce vasorelaxation of precontracted rat aortic tissues [10], scavenge free radicals and superoxide, inhibit lipid peroxidation [12], and fight cancer by inducing apoptosis in the breast cancer cell line MCF-7 [13]. Chemical constituents of *P. aviculare* include flavonoids (avicularin, kaempferol, quercetin, and myricetin), cafferic acid, chlorogenic acid, coumaric acid, oxalic acid, D-catechin, and gallic acid [14]. Phenolics and flavonoids found in *P. aviculare* at high levels exert multiple biological effects, including antioxidant and antitumor activities with the capacity to scavenge free radicals [13]. However, the antiobesity substances within *P. aviculare *have not been assessed until now. In the present study, the effects of a 70% ethanol extract of *P. aviculare *(PAE) on body weight, blood lipid levels, and fat accumulation in high-fat diet-induced (HFD) obese mice and on the differentiation of the murine preadipocytes 3T3-L1 were studied. Additionally, the mRNA expression levels of lipid metabolism-related genes in white adipose tissue were investigated.

## 2. Materials and Methods

### 2.1. Preparation of PAE

*P. aviculare *was purchased as a dried herb from Omniherb Co. (Yeoungcheon, Republic of Korea) and was authenticated based on its microscopic and macroscopic characteristics by the Classification and Identification Committee of the Korea Institute of Oriental Medicine (KIOM). The committee was composed of nine experts in the fields of plant taxonomy, botany, pharmacognosy, and herbology. A voucher specimen (no. JA-38) was deposited at the herbarium of the Department of Herbal Resources Research at the KIOM. The dried aerial parts of *P. aviculare *(100 g) were extracted twice with 70% ethanol using a 2 h reflux extraction, and the extract was concentrated under reduced pressure. The concentrate was filtered, lyophilized, and subsequently stored at 4°C. The yield of the dried extract from starting crude materials was 14.03% (w/w).

### 2.2. Cell Culture and Differentiation

3T3-L1 preadipocytes (ATCC, Rockville, MD, USA) were maintained in Dulbecco's modified Eagle's medium (DMEM) supplemented with 10% calf serum, 100 *μ*g/mL penicillin, and 100 *μ*g/mL streptomycin in a humidified atmosphere of 5% CO_2_ at 37°C. The cells were grown in 12-well plates to confluence. To induce differentiation, 2-day postconfluent cells were stimulated for 2 days with DMEM containing 10% fetal bovine serum (FBS), 0.5 mM 3-isobutyl-1-methylxanthine, 1 *μ*M dexamethasone, and 10 *μ*g/mL insulin (MDI). After this induction, the cells were cultured in DMEM with 10% FBS and 10 *μ*g/mL of insulin for 2 days. Cells were then cultured in DMEM with 10% FBS for 4 days. The medium was changed daily. Various concentrations of PAE were added along with the differentiation medium. On day 8, differentiation was assessed by morphological changes and the Oil Red O staining method. A cytotoxicity test was performed to examine the effect of PAE on the viability of 3T3-L1 cells using the 3-(4,5-dimethylthiazol-2-yl)-2,5-diphenyltetrazolium bromide (MTT) assay [15].

### 2.3. Oil Red O Staining

The differentiated cells were washed twice with phosphate-buffered saline and then fixed with 10% formalin for 30 min. The fixed cells were stained with 0.3% oil red O solution for 1 h. The cells were visualized using an Olympus CKX41 microscope (Olympus, Tokyo, Japan) and photographed at 100x magnification using the Motic image Plus 2.0 program (Motic, Causeway Bay, Hong Kong). To quantify the intracellular lipids, the stained lipid droplets were dissolved in isopropanol (500 *μ*L/well), and the absorbance was measured at 520 nm using a spectrophotometer (BioRad, La Jolla, CA, USA). The optical density of fully differentiated adipocytes was set as 100% relative lipid content.

### 2.4. Animals and Diets

Male 4-week-old C57BL/6J mice were purchased from Daehan Biolink Co. (Eumsung, Korea) and maintained for 1 week prior to experiments. They were housed in an air-conditioned animal room with a 12 h light/12 h dark cycle at 22 ± 1°C and 50 ± 10% humidity. Mice were provided with a laboratory diet and water ad libitum. All experimental protocols involving the use of animals were conducted in accordance with National Institutes of Health (NIH) guidelines and approved by the Committee on Animal Care of the KIOM. To induce obesity, the mice were fed a HFD (Rodent diet D12492, Research diet, New Brunswick, NJ, USA) consisting of 60% fat, 20% protein, and 20% carbohydrate, in accordance with previously published reports [16]. Normal mice were fed a commercial standard chow diet (Orient Bio Inc., Seongnam, Republic of Korea). Orlistat and Bangpoongtongsungsan (BPT) were used as positive control treatments of obesity. The mice were randomly divided into five groups (*n* = 7) that were fed a normal diet (ND), a HFD, a HFD plus PAE (HFD + PAE), a HFD plus Orlistat (HFD + Orlistat), or a HFD plus BPT (HFD + BPT) for 6.5 weeks. PAE or BPT were dissolved in normal saline and orally administered to the mice at 400 mg/kg/day for 6.5 weeks. Orlistat was administered to the mice at 15.9 mg/kg. The ND and HFD control mice were treated with vehicle (normal saline) only. Body weight and food intake were measured twice per week.

### 2.5. Biochemical Parameter Analysis of Blood

At the end of the experimental period, the mice were fasted for 15 h prior to sacrifice. Blood samples were centrifuged at 2000 ×g for 15 min at 4°C, and the serum was stored at −70°C until analysis. Serum levels of triglyceride, total cholesterol, high density lipoprotein (HDL) cholesterol, low density lipoprotein (LDL) cholesterol, alanine aminotransferase (ALT), aspartate aminotransferase (AST), blood urea nitrogen (BUN), and creatinine were analyzed with an automatic analyzer (Hitachi 7080, Hitachi Co., Tokyo, Japan). The concentrations of serum leptin and adiponectin were measured with mouse enzyme-linked immunosorbent assay kits (R&D Systems, Minneapolis, MN, USA), according to the manufacturer's instructions. The absorbance was measured using a microplate spectrophotometer (BioRad, Hercules, CA, USA). The serum malondialdehyde (MDA) level was measured using the thiobarbituric acid reactive substances (TBARS) assay kit (Cayman, Ann Arbor, MI, USA).

### 2.6. Adipose Tissue Weight and Histological Analysis

After blood collection, the white adipose tissues (subcutaneous, epididymal, and retroperitoneal) were removed from the mice and weighed immediately. To stain adipocytes, adipose tissues were fixed in 10% neutral formalin solution for one day and embedded in paraffin. All tissues were cut to a thickness of 6 *μ*m and stained with hematoxylin and eosin. To assess the sizes of the adipocytes, the area of 20 adipocyte cells was measured in representative sections by light microscopy (Olympus BX51, Olympus Optical Co., Tokyo, Japan) and an image analysis program (Image-Pro Plus 5.0, Media Cybernetics, Silver Spring, MD, USA).

### 2.7. Reverse Transcriptase-Polymerase Chain Reaction (RT-PCR)

Tissue samples and cells were homogenized, and total RNA was isolated with the easy-BLUE total RNA extraction kit (Intron, Seoul, Republic of Korea) according to the manufacturer's instructions. To synthesize cDNA, 1 *μ*g total RNA was mixed with premix containing oligo (dT) primer and diethyl pyrocarbonate-treated water in a final volume of 20 *μ*L and incubated at 45°C for 60 min. The reaction was stopped by heat inactivation at 95°C for 5 min. Subsequently, the cDNA was amplified with gene-specific primers using the Taq PCR master mix (QIAGEN, Hilden, Germany) according to the manufacturer's instructions. The 20 *μ*L amplification mixture contained 1 *μ*L cDNA, 10 *μ*L 2 × Taq PCR master mix containing 1.5 mM MgCl_2_ and 0.1 *μ*M of each primer, and water. After a 15 min preincubation at 94°C, PCR amplification was performed for 35 cycles under the following cycling conditions: 30 s denaturation at 94°C, 30 s annealing at 60°C, and 1 min extension at 72°C. Gene-specific primers (Table 1) were designed using Primer3 software based on gene sequences from the GenBank database. The relative expression levels of target genes were normalized to a glyceraldehyde 3-phosphate dehydrogenase (GAPDH) internal control.

### 2.8. Western Blot Analysis

Tissue samples were homogenized, and total protein was extracted with the PRO-PREP protein extraction solution (Intron, Seoul, Republic of Korea) according to the manufacturer's instructions. Proteins (40 *μ*g) were separated by 12% sodium dodecyl sulfate-polyacrylamide gel electrophoresis and transferred onto nitrocellulose membranes. Membranes were blocked with 5% nonfat milk and then incubated with primary antibodies against PPAR-*γ* obtained from Santa Cruz Biotechnology (Santa Cruz, CA, USA) and *β*-actin obtained from Cell Signaling Technology (Beverly, MA, USA) at 4°C overnight. The membranes were then incubated with the corresponding horseradish peroxidase-conjugated secondary antibodies (Cell Signaling) for 1 h at room temperature. Membranes were treated with the ECL detection reagent (Amersham Bioscience, Buckinghamshire, UK), and protein bands were visualized using Las-1000 (Fujifilm, Tokyo, Japan).

### 2.9. High-Performance Liquid Chromatography Analysis

The sample was analyzed by reverse phase-high performance liquid chromatography using Waters Alliance 2695 system (Waters Co., Milford, MA, USA), coupled with a 2996 photodiode array detector. Data processing was carried out using Empower software (Waters Co.). Phenomenex Luna C18 column (250 mm × 4.6 mm; particle size 5 *μ*m, Phenomenex, Torrance, CA, USA) was used as stationary phase. The mobile phase was composed of 0.1% (v/v) trifluoroacetic aqueous solution (A) and acetonitrile (B) using a gradient elution of 15% B at 0–25 min, 35% B at 25–45 min, 100% B at 45-46 min, and 100% B at 46–55 min. Reequilibration duration was 15 min between individual runs. The flow rate was 1.0 mL/min, and the injection volume was 20 *μ*L and the column temperature was kept at 40°C. Identification was based on retention time, UV spectra by comparison with commercial standards. For each compound, peak areas were determined at the wavelength providing maximal UV absorbance. Calibration curves of the standards ranging from 3.1 to 50 *μ*g/mL (5 levels) revealed good linearity with *R* ^2^ values exceeding 0.99 (peak areas versus concentration). Quantitation was performed based on external standards with a mixture of standards of known concentration that were analyzed in duplicate before and after the batch of samples, and the peak areas were used to calculate the sample contents of the compounds. HPLC-grade reagents, acetonitrile, and water were obtained from J. T. Baker (Phillipsburg, NJ, USA).

### 2.10. Statistical Analysis

Differences between groups were assessed by an analysis of variance (ANOVA) followed by Duncan's multiple range test. All data are presented as means ± SEM. Differences were considered significant when the *P* value was less than 0.05.

## 3. Results

### 3.1. 3T3-L1 Preadipocyte Differentiation

**Figure 1:**
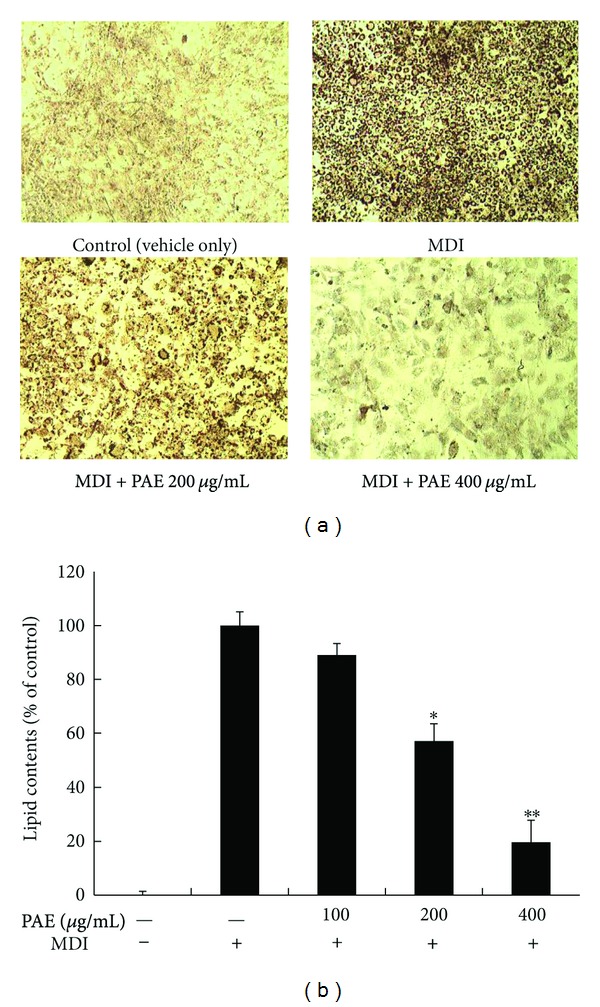
The effect of PAE on 3T3-L1 preadipocyte differentiation. (a) Oil Red-O staining of intracellular triglycerides in 3T3-L1 cells. 3T3-L1 cells were treated with various concentrations (0, 100, 200, and 400 *μ*g/mL) of PAE during the induction of differentiation. (b) Relative density of lipid contents. Values are expressed as means ± SEM (*n* = 3). Cells treated only with MDI are fully differentiated adipocytes. Significant differences were observed between the MDI-only and MDI + PAE-treated cells: *P < 0.05 and **P < 0.01.

Preadipocytes were stained with the fat-specific oil red O at 8 days after the induction of adipocyte differentiation, a point at which they are expected to have reached a fully differentiated state; the state of PAE-treated cells was different from MDI-only stimulated cells that had not been treated with PAE (Figure 1(a)). This observation suggests that adipocyte differentiation was suppressed by the PAE treatment. The quantification of oil red O staining demonstrated that PAE significantly decreased lipid accumulation (Figure 1(b)). PAE did not affect the viability at any concentration tested (data not shown).

### 3.2. Body Weight, Food Intake, and Adipose Tissue Weight

**Figure 2:**
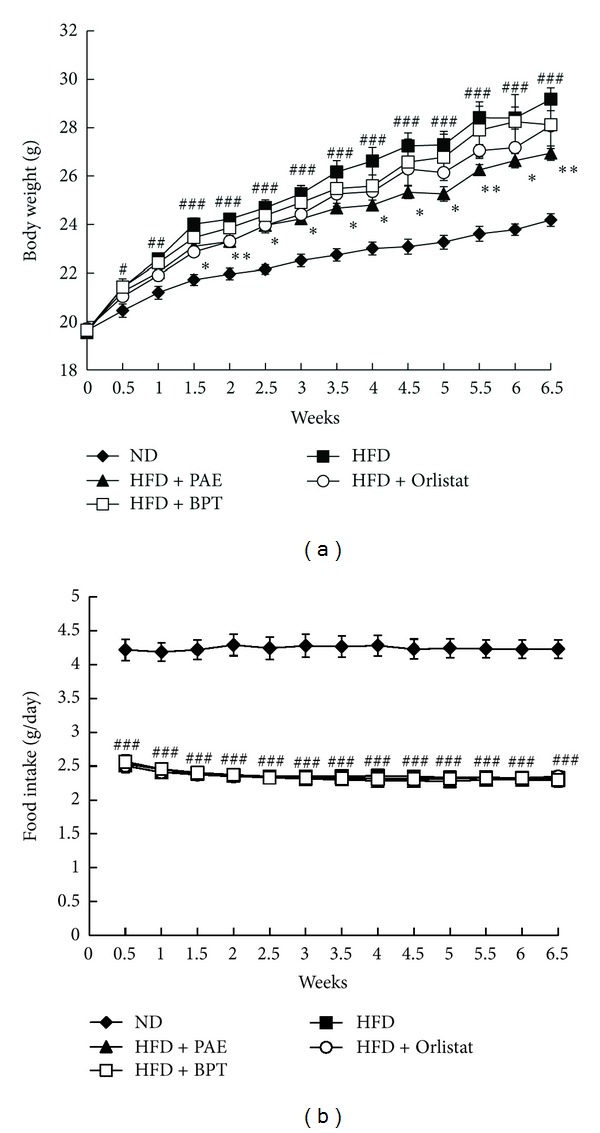
The effect of PAE on body weight and food intake in HFD-induced obese mice. (a) Body weight and (b) food intake. Values are expressed as means ± SEM (*n* = 4). Significant differences were observed between the ND and HFD groups: ^#^ *P* < 0.05 and ^##^ *P* < 0.01 and ^###^ *P* < 0.001. Significant differences were observed between the HFD and the HFD + PAE, HFD + Orlistat, or HFD + BPT groups: *P < 0.05 and **P < 0.01.

The final mean body weight and body weight gain of the HFD group was significantly higher than those of the ND group (Figure 2(a) and Table 2). Six and a half weeks after PAE treatment, the mean body weight and body weight gain of mice were significantly lower than those of mice in the nontreated HFD group (Figure 2(a) and Table 2). During the feeding period, food intake was higher in the ND group than in the HFD group but did not differ significantly between the HFD and HDF + PAE-treated groups; similar results to those observed in the HFP + PAE-treated mice were seen in HFD mice treated with Orlistat or BPT (Figure 2(b) and Table 2).

To investigate whether PAE decreases adiposity, mice were sacrificed, and adipose tissues were removed and weighed. The weights of various white adipose tissues, including subcutaneous, retroperitoneal and epididymal adipose tissues, were increased in the HFD-fed mice relative to the ND-fed mice. The retroperitoneal and epididymal adipose tissue weights were significantly decreased by PAE treatment, but subcutaneous adipose tissue weight did not differ significantly between the HFD and HFD + PAE-treated groups (Table 2). Additionally, the PAE-treated mice did not exhibit significant changes in the weights of the liver or spleen (data not shown).

### 3.3. Histology of Adipose Tissue

**Figure 3:**
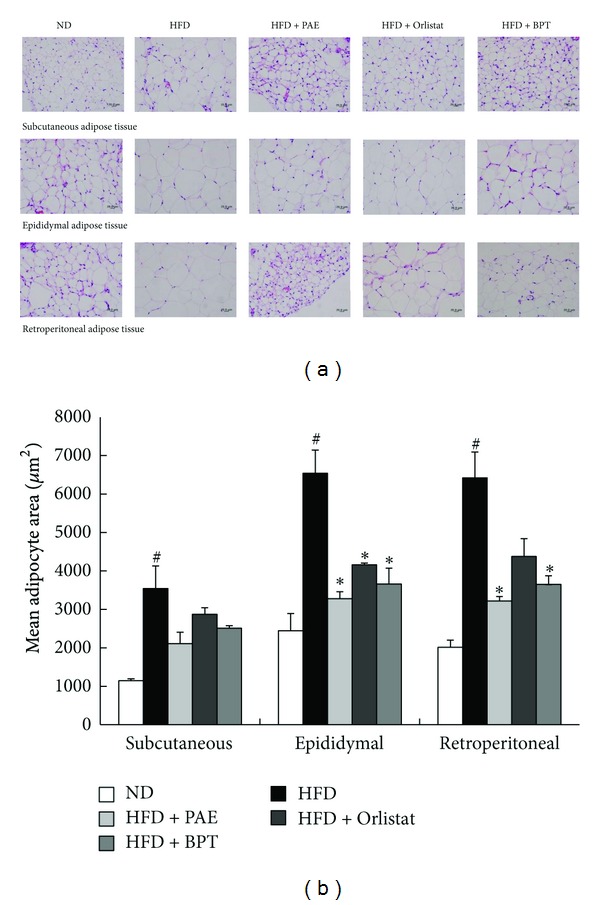
Histology of adipose tissues in HFD-induced obese mice. (a) Representative photographs of hematoxylin and eosin (H&E)-stained sections (magnification, 400x) and (b) adipocyte area. Values are expressed as means ± SEM (*n* = 20). Significant differences were observed between the ND and HFD groups: ^#^ *P* < 0.05. Significant differences were observed between the HFD and the HFD + PAE, HFD + Orlistat or HFD + BPT groups: *P < 0.05.

Stained adipocytes of all the treatment groups are shown in Figure 3(a) and the mean adipocyte areas are shown in Figure 3(b). Adipocytes of the subcutaneous, retroperitoneal, and epididymal adipose tissues were significantly larger in the HFD group than in the ND group. PAE treatment significantly decreased mean adipocyte size relative to that of untreated HFD-fed mice, but the size of subcutaneous adipose tissue adipocytes was not significantly different between the HFD and HFD + PAE-treated groups; similar results to those observed in the HFP + PAE-treated mice were seen in HFD mice treated with Orlistat or BPT (Figure 3(b)).

### 3.4. Serum Biochemical Levels

PAE treatment of HFD-fed mice significantly inhibited the HFD-induced increase in triglyceride levels (Table 3). However, total cholesterol, HDL-cholesterol, and LDL-cholesterol levels did not differ between the HFD and HFD + PAE groups. HFD-fed mice exhibited significantly increased serum leptin levels compared to the levels observed in ND-fed mice (Table 3). However, the HFD + PAE-treated group exhibited decreased serum leptin levels compared to the levels in the HFD group. Serum adiponectin levels did not differ significantly between the HFD and HFD + PAE-treated groups (Table 3).

To evaluate the potential toxic effects of ingesting PAE, serum toxicological markers indicating liver or kidney injury were measured at the end of the experimental period. PAE treatment of HFD-fed mice significantly inhibited the HFD-induced increase in AST levels (Table 3). The levels of ALT, BUN, and creatinine were not significantly changed in HFD + PAE-treated mice compared to the levels in HFD-fed mice (Table 3); similar results to those observed in the HFP + PAE-treated mice were seen in HFD mice treated with Orlistat or BPT. Levels of ALT, BUN, and creatinine of all the treatment groups fell in the normal range according to previously published reports [17, 18]. These data indicate that administration of 400 mg/kg/day PAE for 6.5 weeks induced no detectable adverse toxic effects in the mice.

### 3.5. Expression Levels of Lipogenesis-Related Genes in Adipose Tissue and Differentiated 3T3-L1 Adipocytes

**Figure 4:**
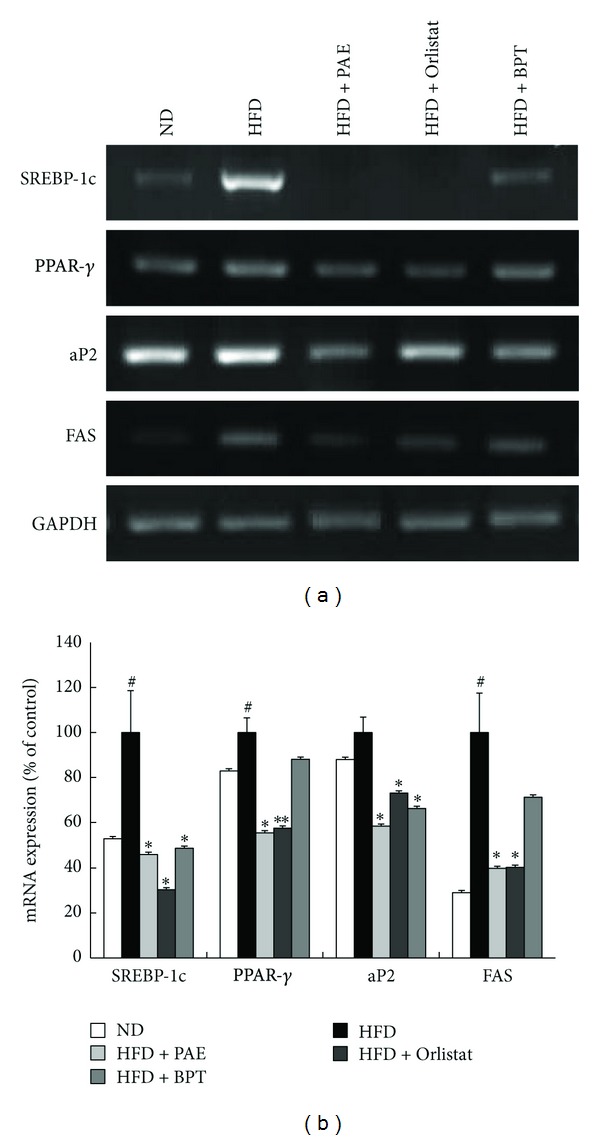
The effect of PAE on gene expression in epididymal adipose tissue of HFD-induced obese mice. (a) Representative bands and (b) relative changes in mRNA expression levels are shown. The relative expression levels of genes were normalized against a GAPDH internal control. Values are expressed as means ± SEM (*n* = 4). Significant differences were observed between the ND and HFD groups: ^#^ *P* < 0.05. Significant differences were observed between the HFD and the HFD + PAE, HFD + Orlistat, or HFD + BPT groups: *P < 0.05 and **P < 0.01.

**Figure 5:**
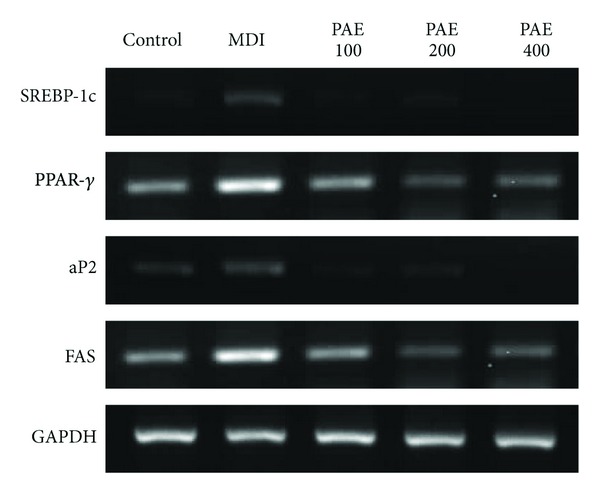
The effect of PAE on gene expression in 3T3-L1 preadipocyte differentiation. 3T3-L1 cells were treated with various concentrations (0, 100, 200, and 400 *μ*g/mL) of PAE during the induction of differentiation. Representative bands in mRNA expression levels are shown.

**Figure 6:**
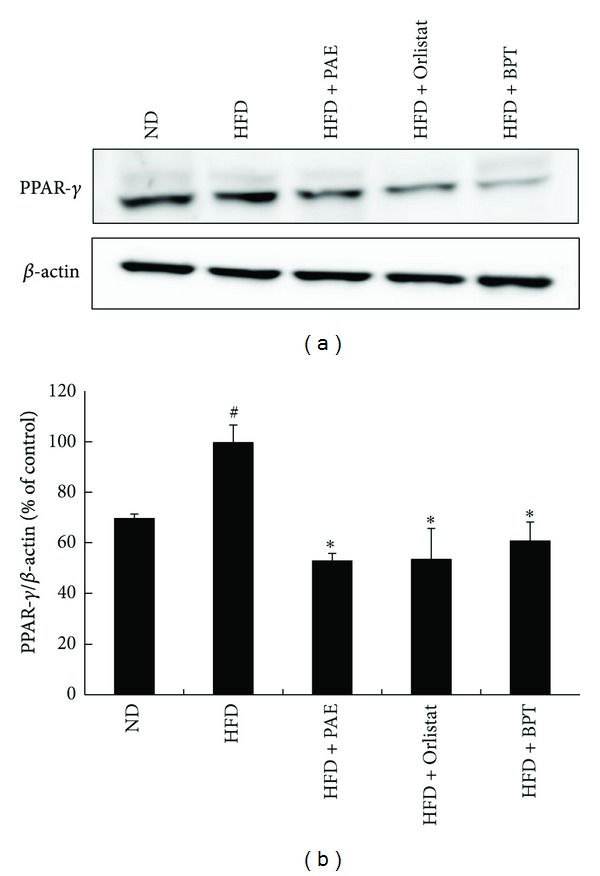
The effect of PAE on protein expression in epididymal adipose tissue of HFD-induced obese mice. (a) Representative bands and (b) relative changes in protein expression levels are shown. The relative expression levels of PPAR-*γ* protein were normalized against a GAPDH internal control. Values are expressed as means ± SEM (*n* = 3). Significant differences were observed between the ND and HFD groups: ^#^ *P* < 0.05. Significant differences were observed between the HFD and the HFD + PAE, HFD + Orlistat, or HFD + BPT groups: *P < 0.05.

To clarify the mechanism involved in the effects of PAE on lipid metabolism, the mRNA expression levels of lipogenesis-related genes in adipose tissue were investigated (Figure 4). Compared with normal mice, the HFD-induced obese mice exhibited increased mRNA levels of adipocyte markers such as sterol regulatory element-binding protein-1c (SREBP-1c), peroxisome proliferator-activated receptor *γ* (PPAR*γ*), and fatty acid synthase (FAS) in the epididymal adipose tissue. However, HFD + PAE treatment significantly decreased the mRNA expression of SREBP-1c, PPAR*γ*, FAS, and adipocyte protein 2 (aP2) as compared with the mRNA levels in the HFD group (Figure 4); similar results to those observed in the HFP + PAE-treated mice were seen in HFD mice treated with Orlistat or BPT. Similarly, treatment with PAE decreased the mRNA expression of SREBP-1c, PPAR*γ*, FAS, and aP2 in differentiated 3T3-L1 cells (Figure 5). Next we performed western blots to determine whether PAE treatment altered the protein levels of PPAR-*γ*, which is involved in lipid synthesis. PPAR-*γ* levels of mice fed a HFD were increased as compared with the ND group and whereas those of HFD + PAE-treated mice were significantly decreased (Figure 6).

### 3.6. Lipid Peroxidation

**Figure 7:**
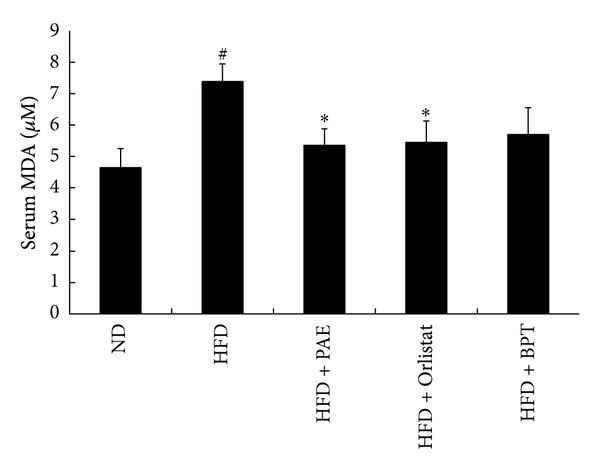
The effect of PAE on serum MDA levels in HFD-induced obese mice. Values are expressed as means ± SEM (*n* = 5–7). Significant differences were observed between the ND and HFD groups: ^#^ *P* < 0.05. Significant differences were observed between the HFD and the HFD + PAE, HFD + Orlistat, or HFD + BPT groups: *P < 0.05.

Lipid peroxidation can be measured by the TBARS assay, which evaluates oxidative stress by assaying levels of MDA, a product of lipid breakdown [19]. Serum MDA was significantly higher in HFD-fed mice than in ND-fed mice, whereas HFD + PAE and HFD + Orlistat treatment significantly inhibited the HFD-induced increase in MDA levels (Figure 7).

### 3.7. Chromatographic Separation

**Figure 8:**
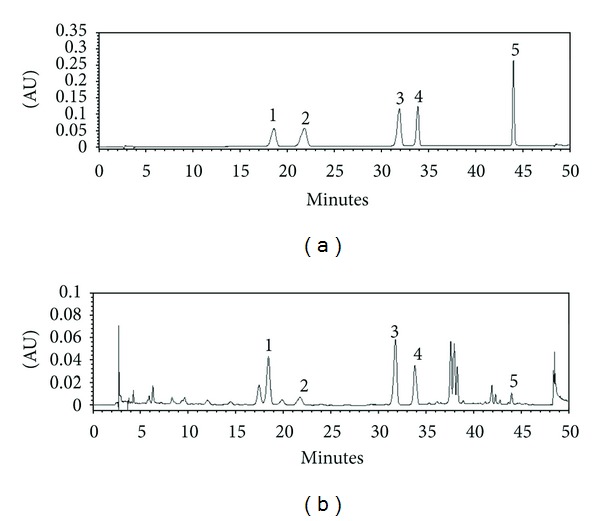
HPLC chromatogram of (a) the standard mixture and (b) PAE at 350 nm. Identification was based on the comparison of retention time and UV spectra to commercial standards. (1) Myricitrin, (2) isoquercitrin, (3) avicularin, (4) quercitrin, and (5) quercetin.

As shown in Figure 8, high performance liquid chromatographic (HPLC) analysis of PAE revealed five standards, myricitrin, isoquercitrin, avicularin, quercitrin, and quercetin at a retention time of approximately 18.5 min, 21.7 min, 31.8 min, 33.8 min, and 44.0 min, respectively. The PAE contained 5.35 ± 0.13 mg/g for myricitrin, 0.96 ± 0.04 mg/g for isoquercitrin, 4.35 ± 0.08 mg/g for avicularin, 2.91 ± 0.05 mg/g for quercitrin, and 0.35 ± 0.02 mg/g for quercetin (Table 4).

## 4. Discussion

Obesity is characterized by increased white fat deposits due to the hyperplasia and hypertrophy of the adipocytes in these deposits. In this study, PAE treatment inhibited white adipose tissue accumulation and the increase in adipocyte size and decreased the weight gain of HFD-induced obese mice. PAE treatment also reduced triglyceride droplet area in differentiated 3T3-L1 cells. These results demonstrate that PAE may reduce adipocyte size by reducing lipid accumulation in these cells, resulting in decreased adipose tissue weight and body weight.

Adipocytes in adipose tissue secrete a variety of proteins known as adipocytokines, including tumor necrosis factor-*α*, interleukin-6, resistin, leptin, and adiponectin [20]. Plasma leptin concentrations are positively correlated with adiposity (excessive body fat) and body weight changes in humans and rodents [21]. Adiponectin contributes to insulin sensitivity and fatty acid oxidation, and circulating concentrations of adiponectin are inversely correlated with body mass [22, 23]. In the present study, serum leptin levels were lower in the HFD + PAE-treated group than in the untreated HFD group. However, serum adiponectin levels were not changed in the HFD + PAE-treated group. These results suggest that the reduction of serum leptin levels with PAE treatment may be attributable to decreased fat accumulation in the adipose tissue and to weight loss.

The adipose tissue is responsible for whole-body energy homeostasis. PPAR*γ*, a transcription factor predominantly expressed in adipose tissue, plays an important role in adipocyte differentiation, lipid storage, and glucose homeostasis [24, 25]. PPAR*γ* regulates the gene expressions of enzymes involved in adipose tissue lipogenesis, such as FAS, aP2, and lipoprotein lipase [26, 27]. FAS is a major lipogenic enzyme that catalyzes the biosynthesis of long chain fatty acids from acetyl-CoA precursors [28]. aP2 is an intracellular fatty acid-binding protein 4 that binds long chain fatty acids [29]. SREBP-1 activates FAS and PPAR*γ* gene expression [30, 31]. In the present study, PAE treatment significantly decreased the mRNA levels of the lipogenesis-related genes PPAR*γ*, SREBP-1c, aP2, and FAS in the adipose tissue of HFD-fed mice and differentiated 3T3-L1 cells. Also, PAE treatment decreased the protein levels of PPAR-*γ* in the adipose tissue of the obese mice. These results suggest that PAE treatment improved intracellular fatty acid metabolism by suppressing adipogenic gene expression.

Reactive oxygen species (ROS) are produced under physiological conditions and have important roles in cell signaling and homeostasis [32]. However, increased levels of ROS due to environmental stress (ultraviolet light, ionizing radiation, chemical reactions, and metabolic processes) cause oxidative stress that significantly damages cell biological systems and is thus involved in pathological processes such as obesity, diabetes, inflammatory disease, cardiovascular disease, and atherogenic processes [33]. ROS also mediate adipocyte differentiation of mesenchymal stem cells [34] and have pathological effects such as DNA damage, lipid peroxidation, protein peroxidation, and cellular degeneration [12]. Accumulation of body fat is correlated with an increase in the lipid peroxidation product, MDA [35]. The quantity of TBARS is an important indicator of lipid peroxide accumulation in the body [36]. The MDA level in serum decreased significantly in the group treated with PAE. The reduction in the lipid peroxidation could be related to the antioxidant and free radical scavenging properties of PAE. Ethanol extracts of *P. aviculare* contain high phenolic and flavonoid levels and exhibit excellent antioxidant activities, scavenging of free radicals and, superoxide radicals, the inhibition of lipid peroxidation, and the prevention of DNA damage [12]. Phenolics exhibit high antioxidant activities and play a preventive role against the development of obesity, cancer, cardiovascular diseases, and neurodegenerative diseases [33, 37]. Thus, this result suggests that the antioxidant activity of PAE may, at least partly, contribute to the reduction of adipose tissue.

According to our HPLC analysis, the ethanol extract of *P. aviculare* contained myricitrin, isoquercitrin, avicularin, quercitrin, and quercetin. Quercetin is a major bioflavonoid that is found in wide variety of vegetables, tea, wine, and fruits. In animal models and cellular lines, quercetin has been reported to have antioxidant and antiobesity effects [38, 39]. It was also reported that quercetin inhibits adipogenesis by activating the AMP-activated protein kinase signal pathway in 3T3-L1 cells [40]. In the present study, we confirmed the presence of quercetin in PAE at a concentration of 0.35%, and this partially explains its antiobesity activity of PAE. However, further study is needed to clarify the pharmacological mechanisms of PAE and to identify the active components responsible for its antiobesity and antioxidant effects.

## 5. Conclusion

In conclusion, the administration of PAE to HFD-induced obese mice significantly reduced body weight gain, adipose tissue weight, adipocyte size, and lipogenic gene expression as well as serum triglyceride, leptin, and MDA levels. Furthermore, PAE inhibited 3T3-L1 adipocyte differentiation. These results suggest that PAE exerts antiobesity effects in HFD-induced obese mice by suppressing lipogenesis in white adipose tissue and through its antioxidant properties. The antiobesity effects of PAE support its potential as a therapeutic or a source of therapeutic substances; its low toxicity in mice and its historical use for other indications in humans indicate it may be safer than antiobesity pharmaceuticals currently available.

## Figures and Tables

**Table 1:** Sequences of primers used in RT-PCR analysis.

Gene	Forward primer (5′-3′)	Reverse primer (5′-3′)	GenBank accession number	Product length (bp)
SREBP-1c	AGCTCAAAGACCTGGTGGTG	TCATGCCCTCCATAGACACA	NM_011480	216
PPAR-*γ*	CCCTGGCAAAGCATTTGTAT	GAAACTGGCACCCTTGAAAA	NM_011146	225
FAS	CGCACCGGCTACCAAGCCAA	GCTTCCCGGGTTGCCCTGTC	NM_007988	266
aP2	TGGAAGCTTGTCTCCAGTGA	GCTCTTCACCTTCCTGTCGT	NM_024406	225
GAPDH	AAGCTGTGGCGTGATGGCCG	TGGGCCCTCAGATGCCTGCT	NM_008084	228

**Table 2:** The effects of PAE on body weight, food intake, and adipose tissue weight in HFD-fed obese mice.

	ND	HFD	HFD + PAE	HFD + Orlistat	HFD + BPT
Initial body weight, g	19.54 ± 0.21	19.64 ± 0.19	19.64 ± 0.21	19.64 ± 0.20	19.64 ± 0.20
Final body weight, g	24.18 ± 0.26	29.17 ± 0.48^###^	26.94 ± 0.30**	28.07 ± 0.17	28.11 ± 0.98
Body weight gain, g/day	4.54 ± 0.16	9.63 ± 0.32^###^	7.31 ± 0.47*	8.37 ± 0.25*	8.49 ± 0.81
Food intake, g/day	4.23 ± 0.13	2.33 ± 0.02^###^	2.30 ± 0.05	2.35 ± 0.03	2.29 ± 0.10
White adipose tissue weight					
Subcutaneous, g/100 g body weight	0.94 ± 0.04	2.85 ± 0.54^##^	2.29 ± 0.36	2.43 ± 0.15	2.92 ± 0.42
Epididymal, g/100 g body weight	1.13 ± 0.14	5.29 ± 0.58^###^	3.73 ± 0.35*	4.19 ± 0.15	4.78 ± 0.81
Retroperitoneal, g/100 g body weight	0.36 ± 0.11	2.22 ± 0.39^##^	1.28 ± 0.13*	1.56 ± 0.13	1.68 ± 0.30

Values are expressed as means ± SEM (*n* = 5–7).

^##^ *P* < 0.01 and ^###^ *P* < 0.001 compared with the ND group; *P < 0.05 and **P < 0.01 compared with the HFD group.

**Table 3:** The effects of PAE on blood parameters in HFD-fed obese mice.

	ND	HFD	HFD + PAE	HFD + Orlistat	HFD + BPT
Total cholesterol, mg/dL	121 ± 2.6	161 ± 2.7^###^	157 ± 4.2	147 ± 4.1*	151 ± 7.8
Triglyceride, mg/dL	68 ± 23.3	83 ± 4.5^##^	53 ± 5.9*	76 ± 9.8	51 ± 4.9**
LDL-cholesterol, mg/dL	4.2 ± 0.5	4.8 ± 0.3	5.2 ± 0.3	4.7 ± 0.2	5.8 ± 0.6
HDL-cholesterol, mg/dL	64.4 ± 1.9	66.6 ± 1.0	68.6 ± 1.4	68.2 ± 2.3	65.9 ± 3.0
ALT, U/L	46.4 ± 2.5	35.0 ± 9.9	23.3 ± 1.8	25.4 ± 1.9	24.7 ± 0.5
AST, U/L	81.2 ± 11.6	141.5 ± 19.3^#^	62.5 ± 8.2*	80.0 ± 11.9	55.1 ± 3.1*
BUN, mg/dL	31.6 ± 1.1	21.4 ± 1.3^###^	22.4 ± 1.2	20.1 ± 1.1	21.0 ± 1.2
Creatinine, mg/dL	0.45 ± 0.0	0.41 ± 0.0	0.44 ± 0.0	0.43 ± 0.0	0.48 ± 0.0
Leptin, ng/mL	4.09 ± 0.6	19.40 ± 3.2^###^	8.88 ± 0.9*	9.17 ± 0.9*	10.87 ± 2.8
Adiponectin, *μ*g/mL	12.27 ± 0.5	13.43 ± 0.4	12.85 ± 0.5	11.51 ± 0.9	12.85 ± 0.5

Values are expressed as means ± SEM (*n* = 5–7).

^#^ *P* < 0.05, ^##^ *P* < 0.01 and ^###^ *P* < 0.001 compared with the ND group. *P < 0.05 and **P < 0.01 compared with the HFD group.

**Table 4:** Average contents of the reference compounds in PAE (*n* = 3).

Compound	Average content in PAE (mg/g)
Myricitrin	5.35 ± 0.13
Isoquercitrin	0.96 ± 0.04
Avicularin	4.35 ± 0.08
Quercitrin	2.91 ± 0.05
Quercetin	0.35 ± 0.02
